# Assessment of Prognostic Factors, Clinical Features Including the Microbiome, and Treatment Outcomes in Patients with Cancer of Unknown Primary Site

**DOI:** 10.3390/cancers16193416

**Published:** 2024-10-08

**Authors:** Karolina Dorobisz, Tadeusz Dorobisz, Katarzyna Pazdro-Zastawny

**Affiliations:** 1 Department of Otolaryngology, Head and Neck Surgery, Wrocław Medical University, Borowska 213, 50-556 Wroclaw, Poland; 2 Department of Vascular, General and Transplantation Surgery, Wroclaw Medical University, Borowska 213, 50-556 Wroclaw, Poland

**Keywords:** microbiome, carcinoma ref unknown primary, head and neck cancer, radiotherapy, radiation-induced mucositis

## Abstract

**Simple Summary:**

Cancer of unknown primary site is a group of cancers in which metastases are found, and the primary tumor is not detected with available diagnostic methods. The aim of this study was to evaluate prognostic factors, clinical features including the microbiome, and treatment outcomes in patients with CUP. Diagnosis and treatment of patients with CUP is difficult due to the lack of consensus on this issue. The clinical value of the influence of the microbiome on the development and treatment of cancer is becoming important. The microbiome may become a marker of response to anticancer treatment and the risk of its complications. Fusobacterium and Porphyromonas seem to be the bacteria most important for the development of cancer, also worsening the prognosis by increasing the risk of complications of radiotherapy and shortening the survival rate of patients. Streptococcus and Lactobacillus seem to be bacteria that reduce the risk of cancer.

**Abstract:**

Introductions: cancer of unknown primary site (CUP) is a heterogeneous group of cancers in which metastases are found, and the primary tumor is not detected with available diagnostic methods. CUP is a disease that has not been fully researched, and its biology is unclear. The clinical characteristics of CUP are variable, but the prognosis of patients is usually unfavorable, and the possibilities of radical treatment are limited. The microbiome is the genes and gene products of microorganisms residing in a human body. In recent years, thanks to the use of next-generation sequencing, it is possible to assess the impact of the microbiome on human body functions. Head and neck cancers, due to the rich microbiome of this area, are influenced by it, and dysbiosis may be a risk factor for the development of cancer. Objective of this work: the aim of this study was to evaluate prognostic factors, clinical features including the microbiome, and treatment outcomes in patients with cancer of unknown primary site. Results: in the study group, increased numbers of bacteria of the phyla *Bacteroides*, *Fusobacteria*, *Bacillota*, *Actinomycetota*, *Actinobacteria*, and *Candidatus* were detected, while *Firmicutes* and *Proteobacteria* were detected in smaller numbers. Independent predictors of CUP occurrence were the following: leukocyte count of at most 6.49 × 10^3^/mm, bacteria from the *Proteobacteria* phylum in the microbiome below 11.6%, *Firmicutes* below 22.1%, and *Actinobacteria* at least 11.0%. Increased numbers of *Porphyromonas* and *Fusobacterium* bacteria were associated with the risk of radiotherapy complications and shortened survival rate. Conclusions: clinical diagnosis and treatment of patients with CUP is complicated and difficult due to the lack of consensus on this issue. Treatment and prognosis of patients with CUP is unsatisfactory. The clinical value of the influence of the microbiome on the development, course, and treatment of cancer is becoming increasingly important. The microbiome may become a marker of response to anticancer treatment and the risk of its complications. Immunity modulation with the microbiome provides opportunities for further research on improving the effectiveness of oncological treatment. *Fusobacterium* and *Porphyromonas* seem to be the bacteria most important for the development of cancer, also worsening the prognosis of patients by increasing the risk of complications of radiotherapy and shortening the survival rate of patients. *Streptococcus* and *Lactobacillus* seem to be bacteria that reduce the risk of cancer, reduce the risk of complications, and improve the prognosis of patients. Total protein deficiency and elevated inflammatory markers are also important predictors of cancer risk.

## 1. Introduction

Cancer of unknown primary site (CUP) is a heterogeneous group of cancers in which metastases are found, and the primary tumor is not detected with available diagnostic methods. CUP is a disease that has not been fully researched, and its biology is unclear. The clinical characteristics of CUP are variable, but the prognosis of patients is usually unfavorable, and the possibilities of radical treatment are limited. Unexplained mechanisms cause regression of the primary tumor, early metastasis, and an aggressive course of the disease. CUP constitutes 3–5% of malignant tumors [1,2]. The incidence in the USA is 7–12 per 100,000 inhabitants [3]. The majority—about 70% of CUP cases—concerns the head and neck region [4]. CUP constitutes 5–10% of all head and neck cancers (HNCs), and 75% of them are squamous-cell carcinomas (HNSCC) [4,5]. Metastases in cervical lymph nodes give rise to suspicion of a primary lesion in the head and neck [6]. The primary lesion is most often found in the palatine tonsil or the base of the tongue and may be associated with infection with the Human Papillomavirus (HPV) [6]. Diagnosis is based on imaging methods—ultrasound (USG), computed tomography (CT), magnetic resonance imaging (MRI), positron emission tomography (PET), determination of tumor markers, and histopathological assessment of the tumor. The histopathological result is very important in the diagnostic process, as it can approximate the location of the primary lesion. During treatment, the primary lesion can be diagnosed in 75% of patients [7,8]. CUP is a diagnostic challenge for radiology and histopathology. It also causes difficulties for surgeons and oncologists who undertake the treatment of these patients.

The microbiome is the genes and gene products of microorganisms residing in the human body [9]. The human microbiome contains 38 trillion microorganisms [10,11]. The presence of the microbiome has been confirmed in tissues that were considered sterile [12]. In recent years, thanks to the use of next-generation sequencing, it is possible to assess the impact of the microbiome on body functions. The influence of the microbiome on pathological conditions such as metabolic disorders, cardiovascular diseases, neurological disorders, cancers, and even mental illnesses such as schizophrenia has been described in numerous publications [13,14,15,16,17,18]. In recent years, the influence of the microbiome on various cancers has been analyzed [19]. The microbiome regulates cellular metabolism, takes an active part in biological processes, and affects the body’s immunity [20]. Dysbiosis is a dysfunction of the microbiome regarding its composition and quantity, causing a decrease in diversity and an increase in pathogenic microorganisms. Microbiome disorders are influenced by diet, chemicals, nicotine, alcohol, antibiotics, hygiene, and stress. HNC is affected by this due to the rich microbiome of this area, and dysbiosis may be a risk factor for cancer development [21]. The microenvironment of chronic inflammation with accompanying dysbiosis becomes immunosuppressive, accelerates tumor growth, and reduces the response to anticancer treatment [22].

## 2. Objective of This Work

The aim of this study was to evaluate prognostic factors, clinical features including the microbiome, and treatment outcomes in patients with cancer of unknown primary site.

## 3. Material and Methods

This study included a group of 40 patients treated for squamous-cell CUP of the neck (11 women and 29 men), aged 47 to 87 years (mean M = 65.6, SD = 8.9 years). This study was conducted at the Department of Otolaryngology, Head, and Neck Surgery of the University Clinical Hospital in Wrocław. Patients were included in this study consecutively. This study was conducted in 2020–2024. The study group was dominated by men (72.5%). The inclusion criteria for this study were patients diagnosed with squamous-cell CUP of the neck treated surgically with adjuvant radio-chemotherapy. The exclusion criteria for this study were patients with chronic inflammation of the upper and lower respiratory tract, treated with antibiotics, probiotics, and other supplements in the last 6 months, other cancers and acute infections, and using elimination diets. Patients were analyzed in the following groups: comparison of the study group with the control group, comparison of patients who did and did not develop radiotherapy-induced mucositis that resulted in delay or inability to complete treatment, and comparison of patients in the study group according to survival rate.

Each patient underwent imaging diagnostics—CT, MRI, PET, and histopathological examination with HPV assessment—and then the disease was classified according to the TNM Classification of Malignant Tumours 8th edition, based on which a decision was made about the method of treatment for the patient [23]. The general condition of the patients was assessed according to the Eastern Cooperative Oncology Group Performance Status Scale (ECOG). Laboratory tests were analyzed for each patient, including blood count and nutritional parameters—total protein and *C*-reactive protein (CRP). The nutritional status of patients was assessed via a BMI assessment and Mini Nutritional Assessment Short FORM (MNA-SF), qualifying them to the group of people with satisfactory nutrition, at risk of malnutrition, or malnutrition [24]. Then, a swab for microbiological culture and a swab for microbiome assessment were taken from each patient before starting treatment, at the diagnostic stage. The microbiome was assessed using 16S rRNA sequencing.

### 3.1. Microbiome Profiling

1. DNA isolation from cotton swab samples using a commercial kit following the manufacturer’s protocol (GeneMATRIX Swab-Extract DNA Purification Kit, Eurx, Gdansk, Poland);
2. Quality control of isolated DNA—concentration and purity evaluation (Qubit 4 Fluorometer, Invitrogen and DeNovix DS-11 spectrophotometer, Guilford, CT, USA); DNA integrity check via electrophoresis on 1.5% agarose gel;
3. Amplifier libraries construction next to rounds of PCR amplification:
  - Amplification of specific target DNA region of bacterial 16S ribosomal RNA (V3–V4) using universal primers connected with Illumina sequencing adapters; PCR Clean-Up using AMPure XP beads, Indianapolis, IN, USA;
  - Index PCR attaching dual indices and Illumina sequencing adapters using the Nextera XT Index Kit, San Diego, CA, USA; PCR Clean-Up using AMPure XP beads, Indianapolis, IN, USA;
4. Library QC, Quantification, normalization, and pooling;
5. Sequencing on MiSeq using paired 300 bp reads.

### 3.2. Control Group

The control group consisted of 30 healthy volunteers aged 45 to 73 years (mean M = 61.4, SD = 8.2). People classified in this group did not suffer from cancer. Exclusion criteria included the same conditions as reported in the study group.

People in the compared groups were homogeneous in terms of sociodemographic characteristics and body mass index (DMI), (*p* > 0.05).

Basic statistics characterizing the patients and healthy controls are presented in Table 1.

### 3.3. Statistical Analysis

Statistica v.13.3 (TIBCO Software Inc., Palo Alto, CA, USA). The compliance of the distribution of quantitative characteristics with the normal distribution in groups was checked using the Shapiro-Wilk test, and the homogeneity of variances was checked using the Bartlett and Levene test. The significance of differences in mean values in two groups of variables with normal distribution and homogeneous variances was checked using the *t* test, and in the case of lack of homogeneity of variances, the t-Welach test was used. The significance of differences in mean values in two groups for features with a non-normal distribution was checked using the non-parametric Mann-Whitney U test. One-way analysis of variance (ANOVA) was used to compare means in more than two groups. If the assumptions of the analysis of variance were not met, the Kruskal-Wallis test was used for comparisons and the Dunn test as a post-hoc test. For all statistical tests, a *p* value < 0.05 was considered significant.

This study was approved by the bioethical committee of Wroclaw Medical University, Poland, 150/22. This study was conducted in accordance with the Declaration of Helsinki, and all participants were informed about the purpose of this study and gave their written consent.

## 4. Results

### 4.1. Analysis of Medical History of the Study Group Compared to the Control Group

The ECOG scale score did not differ between the study and control groups; 82.5% of patients from the control group and 64.7% of patients from the study group were classified as ECOG 0. The remaining patients were classified as ECOG 1—presence of disease symptoms and ability to walk and work. The differences between the groups were not significant. Chronic diseases were more common in the control group—76.7% compared to 50% of the study group patients. Cardiovascular diseases dominated among chronic diseases. Patients with CUP were more likely to suffer from lower respiratory tract diseases; this concerned 17.5% of patients. None of the control group patients reported lower respiratory tract diseases—the difference was statistically significant. In the study and control groups, the number of patients smoking tobacco was comparable—57.5% of CUP and 60% of the control group. In the group of patients with CUP, 12.5% regularly consumed alcohol; in the control group, none of the persons drank alcohol regularly; the difference was not significant. The condition of the teeth was significantly worse in patients with CUP; caries was found in 47.5% of patients, and edentulism was found in 15%. The nutritional status in both groups was satisfactory. The Visual Analogue Scale (VAS) score of 1 was reported by 7.5% of patients in the study group, and VAS 0 was reported in all patients in the control group. The discussed data are presented in Table 2.

### 4.2. Clinical Characteristics of Patients with CUP Syndrome

In the study group, an HPV-positive result was obtained in 20% of patients. The N feature in the TNM scale was assessed as N1 in 55%, N2a in 12.5%, N2b in 22.5%, and N2c in 10% of patients. No distant metastases were observed in patients from the study group. In stage I, there were 12.5% of patients, II—5%, III—42.5%, and IV—40%. Metastases were most frequently detected in group II lymph nodes in the neck (65% of cases); metastatic nodes were detected in 22.5% of patients in group II and in 37.5% of patients in group III. Radiation-induced oral mucositis (RIOM) affected 40% of patients with CUP. Survival rate between 12 and 24 months was observed in 15% of patients, 24–36 months in 20%, 36–48 months in 5%, and more than 48 was obtained in 60% of patients. These data are summarized in Table 3.

### 4.3. Analysis of Laboratory Tests

In blood tests, CUP patients had statistically significantly higher leukocyte levels than the control group. Total protein levels differed significantly between groups; in CUP patients, they were lower and averaged 6.6 g/dL. The CRP level in the study group was significantly higher than in the control group, averaging 4.9 mg/L (3.9–6.3) compared to the control group of 3.2 mg/L (2.1–4.1). These data are presented in Table 4.

### 4.4. Analysis of Bacteria Cultures between the Studies Group and the Control Group

In the study group, Streptococcus oralis was less frequently found in the oral swab culture as a physiological bacterial flora compared to the control group (36.4% vs. 70%); the difference was statistically significant. In the study group, pathogenic bacteria such as Staphylococcus aureus, Candida, Pseudomonas, Serratia, Klebsiella, and others listed in Table 5 were more frequently detected. Candida albicans was more frequently detected in the cultures of patients from the study group; such a result was obtained in 38.6% of patients.

### 4.5. Analysis of the Oral Cavity Microbiome in Patients with CUP Compared to Patients from the Control Group

Table 6 and Table 7 present basic statistics (means and standard deviations) regarding the percentage of isolated bacterial and fungal species in the study and control groups and the results of significance tests. In patients with CUP, significantly more *Prevotella*, *Fusobacterium*, *Actinobacteria*, *Actinomyces*, *Candidadus*, *Clostridiales*, *Veilonella*, *Eikenella*, *Stomatobaculum longum*, *Tannerella*, *Bifidobacteriaceae*, *Enterobacteriaceae*, *Eikenella*, *Catonella*, *Eubacterium*, *Oceanivirga miroungae*, *Slackia*, *Filifactor alocis*, *Lachnoanaerobaculum saburreum*, *Cryptobacterium curtum*, *Dysgonomonas*, *Klebsiella*, *Staphylococcus*, *Shigella*, *Finegoldia*, *Gammaproteobacteria*, and *Moryella indoligenes* were found. In the study group, the number of *Streptococcus oralis*, *Rothia micilaginosa*, *Neiseria*, *Lactobacillales*, *Haemophilus*, and *Pasteurellaceae* was lower than in the control group. These differences were statistically significant.

### 4.6. Microbiome Analysis in Terms of Phylum between Study Group and Control Group

**Figure 1:**
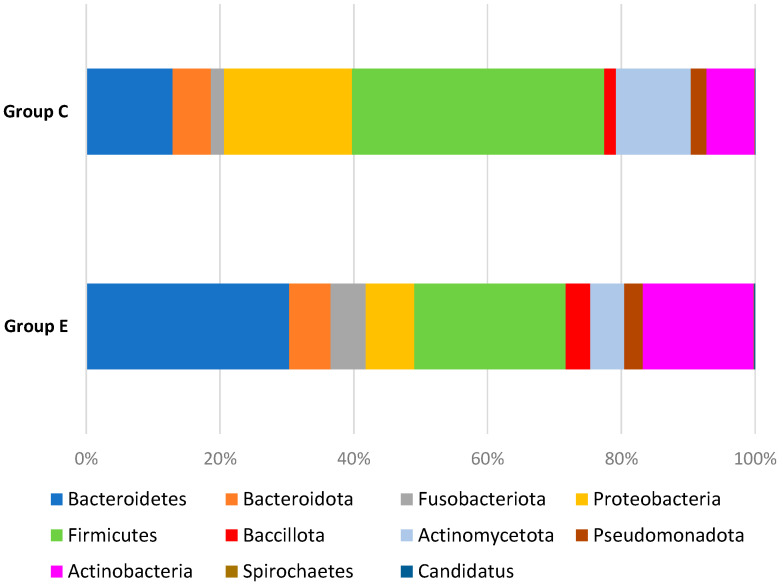
Microbiome of the study and control groups.

In the study group, bacteria from the phyla *Bacteroides*, *Fusobacteria*, *Bacillota*, *Actinomycetota*, *Actinobacteria*, and *Candidatus* dominated; these differences were statistically significant compared to the control group. In the control group, *Firmicutes* and *Proteobacteria* dominated; this difference was statistically significant compared to the study group. These data are presented in Table 8 and Figure 1.

Multivariate logistic regression analysis was used to determine independent factors associated with the occurrence of CUP. The dichotomous dependent variable was the diagnosis of the disease (group S = 1, group C = 0), and the independent variables were clinical parameters from the interview and microbiome assessment. Cut-off values of continuous variables were determined based on the analysis of ROC curves. Independent predictors of CUP occurrence were the following: leukocyte count higher than 6.49 × 10^3^/mm, number of *Proteobacteria* bacteria below 11.6%, *Firmicutes* below 22.1%, and *Actinobacteria* not less than 11.0%. Data are presented in Table 9.

### 4.7. Analysis of the Study Group for the Occurrence of RIOM

No statistically significant difference was observed between patients with different complications after radiotherapy in terms of sociodemographic characteristics (*p* > 0.05, Table 10).

Patients with RIOM had lower total protein level (*p* = 0.036), increased CRP (*p* < 0.001), worse dental status—edentulism, caries, and periodontal disease (*p* < 0.001)—, more frequently positive culture results (*p* < 0.001), more advanced lymph node metastases (*p* = 0.008), higher stage of cancer (*p* = 0.009), and shorter survival rate (*p* < 0.001). Results are presented in Table 11.

### 4.8. Microbiome Analysis Depending on the Occurrence of RIOM

High levels of isolated *Porphyromonas* and *Fusobacterium* were found to be risk factors for radiotherapy complications—RIOM. The risk of complications is significantly higher in patients with isolated *Porphyromonas* bacteria, with a concentrations of at least 2.9%, and *Fusobacterium* bacteria with concentrations of at least 3.2%. These data are presented in Table 12 and Table 13.

Risk factors for RIOM were the number of *Bacteroidota* ≥ 4.8%, *Fusobacteriota* ≥ 3.5%, and Firmicutes ≥ 20.9%. The differences between these groups were statistically significant and are presented in Table 14.

### 4.9. Analysis of the Study Group in Terms of Survival Rate

No statistically significant difference was observed between patients differing in terms of sociodemographic characteristics. These data are presented in Table 15.

Lower total protein level and higher CRP level were found in patients with shorter survival rate; the differences were statistically significant. Shorter survival rate was more common in patients with dental diseases—such as caries, periodontal disease, and with positive oral culture results. Patients with shorter survival rate more often developed RIOM. These data are presented in Table 16.

### 4.10. Analysis of the Microbiome According to Patient Survival Rate

The risk factors for shortened survival rate were high levels of isolated *Porphyromonas* and *Fusobacterium* and reduced levels of *Streptococcus*; these differences were statistically significant. The results are presented in Table 17.

In the analysis of the microbiome by phylum, it was shown that increased amounts of *Bacteroidota* and *Fusobacteriota* increase the risk of shorter survival rate. On the other hand, a larger component of *Firmicutes* promotes longer survival. Data are presented in Table 18.

## 5. Discussion

A neoplasm presenting as a nodal metastasis without a primary site is initially defined as a malignant neoplasm of unknown origin (MUO). After a thorough clinical analysis, the primary focus can be diagnosed in 75% of cases, while in the remaining cases, CUP is diagnosed [8]. Theories on the development of CUP are different, and the pathogenesis of CUP has not yet been clearly explained. Although the process of its development is unclear, an undetectable primary tumor is suspected, or a primary focus undergoing regression, or development from stem cells that have undergone transformation in a given location [25,26]. In their publications, the authors point out the lack of consensus on the diagnosis and treatment of patients with CUP; difficult patient cases published show how complicated the diagnostic and therapeutic process is in CUP [27]. The most common locations of the primary focus are the oral part of the pharynx—the palatine tonsil, base of the tongue, and lingual tonsil [3]. Other locations include the nasopharynx or lower pharynx. Nodal metastases in CUP usually concern groups I, I, and III in the neck, which was also confirmed in the discussed work [3,28]. HPV is often detected in HNSCC CUP, which allows us to suspect the location of the primary origin in the palatine tonsil or the base of the tongue; in the discussed study, HPV positivity was obtained in 20% of patients [29,30]. HPV-positive SCCs produce nodal metastases in the early stages of the disease, when the primary lesion is very small or may be undetectable [31]. According to Weiss et al. [32], 34% of HPV-positive SCCs initially manifest as CUP. The prognosis of patients with HPV-positive head and neck cancers is better than in those negative for HPV. During the diagnostics of CUP, a full radiological assessment, panendoscopy with biopsies, and bilateral tonsillectomy should be performed to try to identify the primary tumor. Resection of the base of the tongue TORS or transoral laser surgery TLM may also be considered—this increases the chances of finding the primary tumor. Detection of the primary tumor is associated with a better prognosis [33]. Post-mortem studies show a primary tumor in 50–80% of CUP [34]. The best treatment for CUP is therapy based on the probable primary tumor. Gene expression profiling increases the chance of identifying the tumor. Due to the lack of a primary tumor, patients often feel anxious about the poor prognosis and risk of recurrence [35]. Cancers are currently often divided into subcategories based on their molecular features [36]. However, molecular classification in CUP syndrome has not yet been established [37]. Treatment of CUP consists of surgery with adjuvant chemoradiotherapy. Immunotherapy could improve the effects of anticancer treatment of CUP, but CUP markers that could indicate patients who will benefit from this therapy have not been verified.

Over the past 40 years, the number of smokers and alcohol consumption has decreased, but this has not resulted in a reduction in the incidence of HNSCC [38]. This may indicate the importance of other risk factors. HNSCC used to affect mainly men, but now women are also increasingly affected, and the average age of patients is also lower. The literature indicates that over 10% of malignant tumors are caused by oncoviruses [39]. These viruses integrate with the host genome, causing mutations—such as HPV in cervical and throat cancer and *hepatitis B* virus (HBV) in liver cancer [40,41]. Up to 25–35% of oral and throat cancers are attributed to HPV [42]. *Epstein Barr virus* (EBV) and *Herpes Simplex virus type 1* (*HSV-1*) are also associated with HNNSCC [43,44]. Viral oncoproteins modulate metabolic pathways and alter gene expression. Some bacteria act in a very similar way, secreting toxins that damage DNA, which affects the aggressiveness of the tumor, resulting in increased mortality [45].

The induction of genomic instability and its mutations is one of the most important risk factors for the development of cancer, which can be caused by the microbiome. The association of the microbiome with cancer has been demonstrated as gene mutations promoting the development of cancer, modulation of the immune system, production of metabolites influencing the development of cancer, the rate of cell proliferation, and response to the treatment [46,47,48]. Disturbance of the immune system by the microbiome consists in the induction of inflammation with simultaneous immunosuppressive effects [49]. In the discussed study, inflammation in patients with CUP was confirmed by an increased number of leukocytes and an increase in CRP compared to the control group; in the study group the number of pathological bacteria in cultures also dominated. Electron microscopy confirmed that bacteria in cancer cells do not have walls [50,51]. The concept of intratumoral microflora has been confirmed in the literature [50]. Studies on the gut microbiome have shown a significant effect of dysbiosis on colon, liver, stomach, and breast cancer [20,51]. The microbiome of oral cancer has also been assessed by various researchers [52,53,54]. The normal oral microbiome consists mainly of aerobes; the number of anaerobes increases with the development of oral cancer. *Fusobacterium* and *Porphyromonas* are considered critical bacteria responsible for the development of cancer [55,56]. Katz et al. [57] confirmed the involvement of *Porphyromonas* in gingival cancer. Chang et al. [58] emphasized the association of *Porphyromonas* with the stage of advancement and the presence of metastases in patients with oral cancer. It was also noted that *Fusobacterium*, *Treponema*, and *Leptotricha* occur more frequently in oral cancer [59]. An increased number of *Prevotella* in tumor tissues was also confirmed [60]. *Candida* albicans has been shown to be present in increased amounts in oral cancer [61]. In the discussed study, the same bacteria and *Candida* were also confirmed in significantly higher amounts in patients with CUP in comparison to the control group in the culture and microbiome. The study showed significant differences between the microbiome of patients despite the presence of the risk factor of smoking in both groups, the study and the control group, and similar observations were made by other authors [62]. This observation makes the microbiome important as a risk factor for cancer. *Fusobacterium* enhances inflammation in the microenvironment [63]. This bacterium protects cancer cells from immune reactions and stimulates cancer development through Toll-like receptors [64,65]. The influence of *Fusobacterium* on colon cancer has been confirmed in numerous studies [66,67]. In induced tongue cancer in mice, the influence of *Fusobacterium* and *Porphyromonas* on the TLR2-OL6-STAT3 axis has been described [68]. *Fusobacterium* induces DNA damage through the Ky70/p53 pathway [65]. *Fusobacterium* promotes cancer cell proliferation, promotes cell invasion, enhances inflammation, and inhibits immune response [69]. FadA, adhesin secreted *by Fusobacterium*, promotes the activation of E-cadherin and β-catenin, which causes DNA damage in mouse CRC cells [70]. The influence of *Fusobacterium* on the immune system through CD11b+ myeloid cells, MDSCs, DCs, CD103+ DCs, and macrophages, which promote tumorigenesis, has been described [71]. It also promotes the accumulation of ROS and the production of pro-inflammatory Il-8, Il-1, and TNF. *Fusobacterium* also increases the transcription of non-coding RNA, which causes changes in the histone detector and tumor formation [72]. Many researchers have confirmed that periodontal disease increases the risk of cancer [73,74]. And periodontitis has a significant impact on the development of oral, esophageal, and pharyngeal cancer [21,75]. *Porphyromonas gingivalis* has been associated with the inhibition of cell apoptosis, activation of proliferation, and induction of inflammation, as well as the production of oncometabolites, i.e., reactive oxygen species, butyrate, and acetaldehyde [76]. The same bacteria were detected in an increased amount of the oral microbiome of patients with CUP in the discussed study. Streptococcus, on the other hand, has a positive effect on the condition of the epithelium, reducing inflammation [63]. Reducing the amount of *Streptococcus* also has a significant effect on the pathogenesis of cancer, and lower amounts of *Streptococcus* have been confirmed in esophageal and stomach cancer [77,78]. *Lactobacilli* have anticancer effects, promote apoptosis, strengthen the immune system, and increase the expression of genes that inhibit cancer development [79]. In the discussed study, patients with CUP had a reduced amount of *Streptococcus* and *Lactobacillus* in the oral microbiome compared to the control group. Reduced amounts of *Streptococcus* was also associated with an increased risk of RIOM and shortened survival rate in patients with CUP.

The microbiome affects the effectiveness of oncological treatment at every stage—surgery, radiotherapy, chemotherapy, and immunotherapy [80]. The intestinal microbiome significantly affects oncological treatment and the response to it, increasing susceptibility to toxic complications of this treatment [13,81]. Modulation of the microbiome affects the effectiveness of anticancer treatment [82]. Antibiotic therapies, by disturbing the flora, may negatively affect the effects of anticancer treatment—chemotherapy and radiotherapy. Broad-spectrum antibiotic therapy disturbs the entire flora, i.e., it also destroys bacteria involved in the formation of vitamins and digestion [67]. On the other hand, combining chemotherapy with appropriate antibiotic therapy can effectively inhibit the multiplication of bacteria in tumors, inhibiting tumor growth, and reducing toxic complications of treatment [83,84]. Fecal microbiota transplantation therapy to change the microbiome may also prove to be a beneficial complement to anticancer treatment. Phages kill oncogenic bacteria, modulate the immune system, and their use in future personalized oncology may improve treatment outcomes [85]. The influence of the microbiome on the effects of immunotherapy has also been repeatedly confirmed [86,87]. A mouse model has shown a relationship between resistance to radiotherapy treatment and the composition of the microbiome [88]. It has been confirmed that *Fusobacterium* influences chemoresistance through regulation of autophagy [89]. Radiotherapy is a very important part of CUP treatment. According to Liu et al. [90] and Tonneau et al. [91], changes in the microbiome during radiotherapy using probiotics, prebiotics, and appropriate antibiotics may be an additional supportive oncological treatment. RIOM is a common complication of head and neck radiotherapy, causing severe pain symptoms due to ulceration, dysphagia, malnutrition, and sometimes resulting in treatment discontinuation. RIOM affects half of the patients treated with chemotherapy and the majority of those treated with radiotherapy due to HNSCC [92]. In the discussed study, a relationship between the risk of RIOM and changes in the microbiome was observed—the dominance of *Porphyromonas* and *Fusobacterium* in the group of patients with severe RIOM. Probiotics have been proven to have a positive effect in preventing RIOM during anticancer treatment [93]. They reduce inflammatory responses through antioxidant defense.

Malnutrition and cachexia are associated with reduced survival rate in cancer patients. Cachexia affects up to 80% of cancer patients. Differences in the microbiome have been demonstrated in patients with cachexia [94]. Protein deficiency despite normal BMI was confirmed in the present study in patients with CUP. Microbiome disturbance with increased participation of *Porphyromonas* and *Fusobacterium* was also associated with reduced survival rate in the present study.

### Limitations of the Study and Suggestions for Future Research

It is worth assessing the impact of microbiome modulation as a result of strengthening or weakening oncological treatment. As well as its impact on the quality and length of life of oncological patients. Research on the impact of probiotics and fecal microbiota transplantation on the microbiome and the course of cancer seems to be important and promising for patients with CUP. Understanding the relationship between the microbiome and the development and course of cancer can provide very important information on treatment and prognosis, especially in the difficult to diagnose and treat CUP syndrome. Microbiome research can help improve the quality and effectiveness of cancer treatment.

## 6. Conclusions

Diagnosing and treating patients with CUP is complicated and difficult due to the lack of consensus. The treatment and prognosis of patients with CUP is unsatisfactory. The clinical value of the influence of the microbiome on the development, course, and treatment of cancer is becoming increasingly important. The microbiome may become a marker of response to anticancer treatment and the risk of its complications. Modulation of immunity with the microbiome provides opportunities for further research into improving the effectiveness of oncological treatment. *Fusobacterium* and *Porphyromonas* appear to be the species most relevant to the development of cancer, also worsening patient prognosis by increasing the risk of radiotherapy complications and shortening patient survival rate. *Streptococcus* and *Lactobacillus* appear to be bacteria that reduce the risk of cancer, reduce the risk of complications, and improve patient prognosis. Total protein deficiency and elevated inflammatory markers are important predictors of cancer risk, its unfavorable course, and its prognosis.

## Figures and Tables

**Table 1:** General characteristics of patients in the study and control groups and the results of significance and independence tests.

Variable	Study Group (S) *n*= 40	Control Group (C) *n* = 30	S vs. C *p*-Value
Gender:			0.577 ^a^
Male, n (%)	29 (72.5%)	19 (63.3)	
Female, n (%)	11 (27.5%)	11 (36.7%)	
Age (years):			0.628 ^b^
M (SD)	65.6 (8.9)	62.6 (6.8)	
Education:			0.872 ^a^
Primary	1 (2.5%)	1 (3.3%)	
Secondary	18 (45.0%)	16 (53.4%)	
Incomplete higher	11 (27.5%)	6 (20.0%)	
Higher	10 (25.0%)	7 (23.3%)	
Place of residence:			0.429 ^a^
Village	8 (20.0%)	8 (26.7%)	
Town up to 20,000	7 (17.5%)	2 (6.7%)	
21,000–50,000 inhabitants	8 (20.0%)	4 (13.3%)	
Over 50,000	17 (42.5%)	16 (53.3%)	
Economic zone/urban area:			0.188 ^a^
Yes	31 (77.5%)	18 (60.0%)	
No	9 (22.5%)	12 (40.0%)	
Marital status:			0.951 ^a^
Single	2 (5.0%)	3 (10.0%)	
Partner/married relationship	30 (75.0%)	23 (76.7%)	
With family support	8 (20.0%)	4 (13.3%)	
BMI (kg/m^2^):			
Me [Q1–Q3]	23.7 [22.9–24.6]	23.4 [22.4–25.1]	0.458 ^c^

*n*—number, (%)—percentile, M—mean, SD—standard deviation, Me—median, Q1–Q3—lower and upper quartile, *p*—test significance level, ^a^—Pearson’s chi-square test, ^b^—Student’s *t* test, ^c^—Mann–Whitney U test.

**Table 2:** Number (percentage) of people differing in the presence of cancer and analyzed clinical features, as well as the results of independence and significance tests.

Variable	Group S N = 40	Group C N = 30	S vs. C *p*-Value
ECOG scale (score):			0.528
0—asymptomatic, n (%)	33 (82.5%)	22 (64.7%)	
1—symptomatic, but completely ambulatory, n (%)	7 (17.5%)	8 (21.1%)	
Swallowing disorders (yes)	0 (0.0%)	0 (0.0%)	1.000
Chronic diseases (yes)	20 (50.0%)	23 (76.7%)	0.043
Arterial hypertension, cardiovascular diseases	20 (50.0%)	23 (76.7%)	0.043
Diabetes	1 (2.5%)	0 (0.0%)	1.000
Allergy	0 (0.0%)	0 (0.0%)	1.000
Lower respiratory tract diseases	7 (17.5%)	0 (0.0%)	0.017
Chronic pharyngitis	0 (0.0%)	0 (0.0%)	1.000
Chronic sinusitis	0 (0.0%)	0 (0.0%)	1.000
Chronic diseases of the upper respiratory tract	0 (0.0%)	0 (0.0%)	1.000
Smoking	23 (57.5%)	18 (60.0%)	0.972
Drinking alcohol regularly	5 (12.5%)	0 (0.0%)	0.066
Gastroesophageal reflux disease	0 (0.0%)	0 (0.0%)	1.000
HPV vaccination	0 (0.0%)	0 (0.0%)	1.000
Antibiotic therapy within 6 months	0 (0.0%)	0 (0.0%)	1.000
Dental condition:			<0.001
1—Normal	15 (37.5%)	29 (83.3%)	
2—Cavities, caries, periodontal disease	19 (47.5%)	3 (10.00%)	
3—Edentulism	6 (15.0%)	2 (6.7%)	
Nutritional status:			1.000
1—Satisfactory	40 (100.0%)	30 (100.0%)	
VAS (score)			0.255
0	37 (92.5%)	30 (100.0%)	
1	3 (7.5%)	0 (0.0%)	

**Table 3:** Clinical characteristics of patients with head and neck cancer.

Variable	N = 40 (100%)
HPV+/p16+:	
CUP	8 (20.0%)
Non-viral related CUP	32 (80.0%)
Tumor:	
Tx	40 (100.0%)
Node:	
N1	22 (55.0%)
N2a	5 (12.5%)
N2b	9 (22.5%)
N2c	4 (10.0%)
M0	40 (100.0%)
Stage:	
I	5 (12.5%)
II	2 (5.0%)
III	17 (42.5%)
IVa	16 (40.0%)
Cervical lymph node groups *:	
I	9 (22.5%)
II	26 (65.0%)
III	15 (37.5%)
RIOM:	16 (40.0%)
Survival rate	
12–24 months	6 (15.0%)
24–36 months	8 (20.0%)
36–48 months	2 (5.0%)
more than 48 months	24 (60.0%)

* Multiple choice question, percentages do not add up to 100.

**Table 4:** Clinical characteristics of patients in the study group.

Variable, Me [Q1–Q3]	Group S N = 40	Group C N = 30	S vs. C *p*-Value
Hemoglobin (g/dL)	13.1 [12.2–14.1]	13.5 (12.8–14.6)	0.089
Leukocytes (×10^3^/mm)	7.8 [7.0–8.8]	5.1 [4.3–6.3]	<0.001
Total protein (g/dL)	6.6 [6.4–6.9]	7.3 [7.1–7.8]	<0.001
CRP (mg/L)	4.9 [3.9–6.3]	3.2 [2.1–4.1]	<0.001

**Table 5:** Number (percentage) of people differing in the presence of cancer and isolated types of bacteria and fungi, as well as in the results of independence tests (Fisher’s exact test).

Culture Result—Genus (Positive)	Group S N = 40	Group C N = 30	S vs. C *p*-Value
*Streptococcus Oralis*	16 (36.4%)	21 (70.0%)	0.009
*Staphylococcus aureus*	4 (9.1%)	0 (0.0%)	0.142
*Candida albicans*	17 (38.6%)	5 (16.7%)	0.069
*Neisseria*	4 (9.1%)	4 (13.3%)	0.707
*Pseudomonas*	5 (11.4%)	0 (0.0%)	0.076
*Serratia mercescens*	3 (6.8%)	0 (0.0%)	0.276
*Bifidobacterium longum*	2 (4.6%)	0 (0.0%)	0.511
*Corynebacterium*	1 (2.3%)	0 (0.0%)	1.000
*Enterococcus faecalis*	1 (2.3%)	0 (0.0%)	1.000
*Klebsiella*, *Enterobacter*, and *Serratia*	3 (6.8%)	0 (0.0%)	0.276
*Citrobacter freundii*	1 (2.3%)	0 (0.0%)	1.000
*Lacticaseibicillus paracasei*	2 (4.6%)	0 (0.0%)	0.511
*Morganella morganii*	2 (4.6%)	0 (0.0%)	0.511
*Streptococcus dysgalactiae*	1 (2.3%)	0 (0.0%)	1.000
*Proteus*	0 (0.0%)	0 (0.0%)	1.000
*Enterobacter cloacae*	0 (0.0%)	0 (0.0%)	1.000
*Veillonella parvula*	1 (2.3%)	0 (0.0%)	1.000
*Escherichia coli*	1 (2.3%)	0 (0.0%)	1.000
Absent	10 (22.7%)	9 (30.0%)	0.590

**Table 6:** Descriptive statistics (mean and SD) of the percentage of isolated bacteria and fungi in the study and control groups and the results of significance tests (Welch’s *t*-test).

Culture Result—Genus (%)	Group S	Group C	*p*-Value
*Streptococcus Oralis*	19.08 (5.24)	31.60 (19.32)	0.002
*Prevotella*	28.11 (6.17)	5.93 (5.12)	<0.001
*Rothia micilaginosa*	3.53 (2.27)	10.01 (6.66)	<0.001
*Aggregatibacter*	1.81 (1.33)	1.63 (4.66)	0.833
*Gemella*	1.45 (1.33)	1.90 (1.47)	0.193
*Porphyromonas*	2.78 (2.87)	3.33 (3.77)	0.512
*Fusobacterium*	3.38 (2.60)	1.28 (1.36)	<0.001
*Corynebacterium matruchotii*	1.60 (1.38)	1.08 (1.61)	0.160
*Neiseria*	3.09 (1.61)	7.06 (7.92)	0.011
*Kingella*	0.77 (0.73)	0.80 (3.09)	0.951
*Lacto bacillales*	0.25 (0.38)	3.10 (2.27)	<0.001
*Actinobacteria*	7.82 (3.46)	2.08 (2.48)	<0.001
*Actinomyces*	5.95 (3.94)	3.85 (4.81)	0.049
*Haemophilus*	2.67 (1.22)	8.53 (7.38)	<0.001
*Capnocytophaga granulosa/gingivalis*	2.33 (1.89)	2.03 (2.73)	0.618
*Treponema*	0.06 (0.14)	0.08 (0.30)	0.727
*Candidadus*	0.12 (0.15)	0.01 (0.04)	<0.001
*Clostridiales*	0.73 (0.70)	0.21 (0.33)	<0.001
*Veilonella*	0.62 (1.21)	0.09 (0.13)	0.009
*Campylobacter*	1.37 (1.02)	1.42 (1.44)	0.857
*Granulicatella*	0.09 (0.15)	0.37 (1.77)	0.392
*Cloacibacterium*	0.03 (0.08)	0.15 (0.66)	0.341
*Lautropia*	1.37 (2.04)	0.66 (1.15)	0.070
*Abiotrophia defective*	0.14 (0.28)	0.02 (0.13)	0.029
*Eikenella corrodens*	0.23 (0.37)	0.12 (0.45)	0.254
*Shaalia odontolytica*	0.65 (0.74)	0.90 (1.38)	0.377
*Leptotricha*	1.47 (2.64)	0.68 (1.09)	0.094
*Stomatobaculum longum*	0.87 (1.08)	0.09 (0.19)	<0.001
*Granucicatella elegans*	0.10 (0.27)	0.91 (2.94)	0.141
*Tannerella*	0.30 (0.54)	0.08 (0.18)	0.021
*Pasteurellaceae*	0.36 (0.57)	0.85 (1.16)	0.040
*Bifidobacteriaceae*	0.14 (0.19)	0.04 (0.17)	0.032
*Atopobium*	0.08 (0.20)	0.04 (0.20)	0.372
*Oribacterium*	0.07 (0.22)	0.00 (0.00)	0.119
*Enterobacteriaceae*	1.84 (2.01)	0.00 (0.00)	<0.001
*Parascardovia denticolens*	0.04 (0.11)	0.00 (0.00)	0.093
*Butyrivibrio*	0.02 (0.06)	0.00 (0.00)	0.057
*Eikenella*	0.37 (0.72)	0.00 (0.00)	0.007
*Cardiobacterium hominis*	0.05 (0.19)	0.00 (0.00)	0.159
*Bergeyella cardium*	0.19 (0.54)	0.00 (0.00)	0.056
*Catonella*	0.16 (0.38)	0.00 (0.00)	0.024
*Vallitalae okinawensis*	0.02 (0.04)	0.00 (0.00)	0.027
*Olsenella*	0.16 (0.46)	0.00 (0.00)	0.061
*Mogibacterium*	0.04 (0.08)	0.00 (0.00)	0.009
*Eubacterium*	0.20 (0.48)	0.00 (0.00)	0.027
*Sulfurihydrogenibium*	0.09 (0.34)	0.00 (0.00)	0.160

**Table 7:** Descriptive statistics (mean and SD) of the percentage of isolated rare bacteria and fungi in the study and control groups and the results of significance tests (Welch’s *t*-test).

Culture Result—Genus (%)	Group S	Group C	*p*-Value
*Oceanivirga miroungae*	0.03 (0.08)	0.00 (0.00)	0.034
*Vallitalea okinawesis*	0.01 (0.04)	0.00 (0.00)	0.154
*Peptostreptococcus anaerobius*	0.28 (0.84)	0.00 (0.00)	0.076
*Mycoplasma salivarius/faucium*	0.06 (0.19)	0.00 (0.00)	0.094
*Dialister pneumosintes*	0.04 (0.17)	0.00 (0.00)	0.277
*Pseudoramibacter alactolyticus*	0.01 (0.03)	0.00 (0.00)	0.129
*Slackia*	0.03 (0.05)	0.00 (0.00)	0.004
*Filifactor alocis*	0.04 (0.07)	0.00 (0.00)	0.011
*Gordonibacter*	0.01 (0.03)	0.00 (0.00)	0.129
*Lachnoanaerobaculum saburreum*	0.02 (0.04)	0.00 (0.00)	0.015
*Cryptobacterium curtum*	0.03 (0.07)	0.00 (0.00)	0.018
*Scardovia wiggsiae*	0.07 (0.31)	0.00 (0.00)	0.201
*Dysgonomonas*	0.06 (0.14)	0.00 (0.00)	0.027
*Parvimonas*	0.02 (0.07)	0.00 (0.00)	0.116
*Ihubacter*	0.02 (0.06)	0.00 (0.00)	0.057
*Phocaeicola abscessus*	0.01 (0.03)	0.00 (0.00)	0.129
*Marinifilum*	0.13 (0.76)	0.00 (0.00)	0.361
*Klebsiella*	0.03 (0.07)	0.00 (0.00)	0.020
*Staphylococcus*	0.12 (0.29)	0.00 (0.00)	0.022
*Anaerococcus*	0.03 (0.10)	0.00 (0.00)	0.094
*Eschericia coli*	0.08 (0.25)	0.00 (0.00)	0.088
*Salmonella*	0.01 (0.05)	0.00 (0.00)	0.190
*Shigella*	0.11 (0.26)	0.00 (0.00)	0.023
*Finegoldia*	0.03 (0.06)	0.00 (0.00)	0.014
*Lawsonella clevelandensis*	0.02 (0.04)	0.00 (0.00)	0.059
*Ottovia*	0.04 (0.13)	0.00 (0.00)	0.129
*Johsonella*	0.03 (0.10)	0.00 (0.00)	0.132
*Morococcus cerebrosus*	0.21 (1.25)	0.00 (0.00)	0.361
*Actinobaculum*	0.02 (1.25)	0.00 (0.00)	0.361
*Gammaproteobacteria*	0.02 (0.04)	0.00 (0.00)	0.015
*Moryella indoligenes*	0.02 (0.04)	0.00 (0.00)	0.027
*Pseudoramibacter alactolyticus*	0.01 (0.03)	0.00 (0.00)	0.076
Other genus	1.82 (1.49)	9.08 (6.83)	<0.001

**Table 8:** Dneral characteristics of patients in groups with different survival rate and results of signifi tests (Welch’s *t*-test).

Culture Result—Phylum (%)	Group S	Group C	*p*-Value
*Bacteroidetes*	28.11 (6.17)	12.69 (9.55)	<0.001
*Bacteroidota*	5.72 (3.82)	5.59 (5.56)	0.914
*Fusobacteriota*	4.88 (4.17)	1.95 (1.70)	<0.001
*Proteobacteria*	6.71 (3.13)	18.76 (12.36)	<0.001
*Firmicutes*	20.97 (5.51)	36.99 (19.90)	<0.001
*Baccillota*	3.38 (1.94)	1.70 (3.33)	0.018
*Actinomycetota*	4.74 (2.49)	10.99 (7.32)	<0.001
*Pseudomonadota*	2.55 (2.58)	2.31 (3.26)	0.737
*Actinobacteria*	15.37 (5.36)	7.01 (6.94)	<0.001
*Spirochaetes*	0.06 (0.14)	0.08 (0.30)	0.727
*Candidatus*	0.12 (0.15)	0.01 (0.04)	<0.001

**Table 9:** Results of univariate and multivariate logistic regression analysis.

Variable	Univariate Analysis			Multivariate Analysis		
	b	*p*	OR	Beta	*p*	OR (95% CI)
Leukocytes ≥ 6.49	2.740	<0.001	15.5	3.248	0.037	25.7 (1.23–540)
Total protein ≤ 6.9	3.109	<0.001	22.4	0	>0.05	1.00
CRP ≥ 4.87	30.05	0.001	67.3			
Chronic diseases	1.190	0.030	3.29	0	>0.05	1.00
*Bacteroidetes* ≥ 20.6%	3.387	<0.001	29.6	0	>0.05	1.00
*Fusobacteriota* ≥ 3.3%	1.809	0.001	6.10	0	>0.05	1.00
*Proteobacteria* ≤ 11.6%	4.210	<0.001	67.4	4.271	0.011	71.6 (2.74–1872)
*Firmicutes* ≤ 28.9%	6.368	<0.001	38.0	5.303	0.009	201 (4.05–9951)
*Baccillota* ≥ 1.2%	3.360	<0.001	28.8	0	>0.05	1.00
*Actinomycetota* ≤ 8.6%	2.981	<0.001	18.5	0	>0.05	1.00
*Actinobacteria* ≥ 11.0%	3.584	<0.001	36.0	3.298	0.028	27.1 (1.44–510)
*Candidatus* ≥ 0.1%	2.739	0.001	15.5	0	>0.05	1.00

**Table 10:** General characteristics of patients in groups with different radiotherapy complications and results of significance and independence tests.

Variable	RIOM—Radiation-Induced Oral Mucositis		*p*-Value
	Yes N = 16	No N = 24	
Gender:			0.473 ^a^
Male. n (%)	13 (81.2%)	16 (66.7)	
Female. n (%)	3 (18.8%)	8 (33.3%)	
Age (years):			0.095 ^b^
M (SD)	66.4 (9.4)	61.6 (8.3)	
Education:			0.351 ^a^
Primary	10 (41.7%)	4 (20.0%)	
Secondary	12 (50.0%)	13 (65.0%)	
Incomplete higher	0 (0.0%)	1 (5.0%)	
Higher	2 (8.3%)	2 (10.0%)	
Place of residence:			0.290 ^a^
Village	3 (18.8%)	5 (20.8%)	
Town up to 20,000	4 (25.0%)	3 (12.5%)	
21,000–50,000 inhabitants	1 (6.2%)	7 (29.2%)	
Over 50,000	8 (50.0%)	9 (37.5%)	
Economic zone/urban area:			0.717 ^a^
Yes	13 (81.2%)	18 (75.0%)	
No	3 (18.8%)	6 (25.0%)	
Marital status:			0.153 ^a^
Single	2 (12.5%)	0 (0.0%)	
Partner/married relationship	12 (75.0%)	18 (75.0%)	
With family support	2 (12.5%)	6 (25.0%)	
BMI (kg/m^2^):			
Mean (SD)	23.5 (1.1)	24.3 (2.1)	0.108 ^c^

^a^—Pearson’s chi-square test. ^b^—Student’s *t* test. ^c^—*t*-test for independent sample.

**Table 11:** Clinical characteristics of patients in groups with different radiotherapy complications and results of significance and independence tests.

Variable. Me [Q1–Q3]	RIOM—Radiation-Induced Oral Mucositis		*p*-Value
	Yes N = 16	No N = 24	
Hemoglobin (g/dL)	12.9 [12.2–14.1]	13.4 [12.1–14.1]	0.772
Leukocytes (×10^3^/mm)	8.4 [7.4–9.5]	7.4 [6.8–8.3]	0.109
Total protein (×10^3^/μL)	6.5 [6.3–6.9]	6.9 [6.5–7.1]	0.036
CRP (mg/L)	6.7 [5.6–7.2]	4.0 [3.0–4.9]	<0.001
ECOG scale (score):			1.000
0—asymptomatic	13 (81.2%)	20 (83.3%)	
1—completely ambulatory	3 (18.8%)	4 (16.7%)	
Chronic diseases (yes)	9 (56.3%)	11 (45.8%)	0.747
Arterial hypertension, cardiovascular diseases	9 (56.3%)	11 (45.8%)	0.747
Diabetes	1 (6.2%)	0 (0.0%)	0.400
Lower respiratory tract diseases	5 (31.2%)	2 (8.3%)	0.094
Smoking	10 (62.5%)	13 (54.2%)	0.845
Drinking alcohol regularly	1 (6.2%)	4 (16.7%)	0.631
Dental condition:			<0.001
1—Normal	1 (6.2%)	14 (58.4%)	
2—Cavities, caries, periodontal disease	14 (87.6%)	5 (20.8%)	
3—Edentulism	1 (6.2%)	5 (20.8%)	
VAS (score)			0.553
0	14 (87.5%)	23 (95.8%)	
1	2 (12.5%)	1 (4.2%)	
Positive culture result	13 (81.3%)	1 (4.2%)	<0.001
HPV+/p16+:			0.439
CUP	2 (12.5%)	6 (25.0%)	
Non-viral related CUP	14 (87.5%)	18 (75.0%)	
Node:			0.008
N1	4 (25.0%)	18 (75.0%)	
N2a	2 (12.5%)	3 (12.5%)	
N2b	7 (43.8%)	2 (8.3%)	
N2c	3 (18.7%)	1 (4.2%)	
Staging:			0.009
I	0 (0.0%)	5 (20.8%)	
II	2 (12.5%)	0 (0.0%)	
III	4 (25.0%)	13 (54.2%)	
Iva	10 (62.5%)	6 (25.0%)	
Cervical lymph node groups *:			
I	3 (18.8%)	6 (25.0%)	0.717
II	13 (81.2%)	13 (54.2%)	0.101
III	7 (43.8%)	8 (33.3%)	0.527
Survival rate:			<0.001
12–24 months	5 (31.3%)	1 (4.2%)	
24–36 months	8 (50.0%)	0 (0.0%)	
36–48 months	2 (12.5%)	0 (0.0%)	
more than 48 months	1 (6.2%)	23 (95.8%)	

* Multiple choice question, percentages do not add up to 100.

**Table 12:** Basic statistics (mean and standard deviation) of bacterial concentration in groups of patients with different risk of complications and results of significance tests.

Culture Result—Genus (%)	RIOM—Radiation-Induced Oral Mucositis		*p*-Value
	Yes	No	
*Streptococcus Oralis*	17.16 (4.56)	20.36 (5.47)	0.057
*Prevotella*	27.84 (6.70)	28.29 (5.93)	0.825
*Rothia micilaginosa*	2.90 (2.45)	3.95 (2.09)	0.154
*Aggregatibacter*	1.68 (1.15)	1.90 (1.46)	0.616
*Gemella*	0.99 (0.91)	1.75 (1.49)	0.077
*Porphyromonas*	5.54 (2.61)	0.94 (0.81)	<0.001
*Fusobacterium*	5.69 (1.92)	1.84 (1.69)	<0.001
*Corynebacterium matruchotii*	1.21 (0.98)	1.87 (1.56)	0.140
*Neiseria*	3.03 (1.87)	3.13 (1.44)	0.847
*Kingella*	0.61 (0.65)	0.88 (0.77)	0.259
*Lacto bacillales*	0.33 (0.45)	0.19 (0.33)	0.266
*Actinobacteria*	7.54 (3.14)	8.00 (3.71)	0.691
*Actinomyces*	5.95 (4.06)	5.95 (3.95)	0.997
*Haemophilus*	2.15 (1.39)	3.02 (0.97)	0.039
*Capnocytophaga granulosa/gingivalis*	2.19 (1.82)	2.42 (1.97)	0.712
*Treponema*	0.04 (0.09)	0.08 (0.17)	0.372
*Candidadus Sacharimonas aalborgensis*	0.12 (0.14)	0.13 (0.16)	0.901
*Clostridiales*	0.61 (0.55)	0.80 (0.79)	0.389
*Veilonella*	0.36 (0.74)	0.79 (1.43)	0.279
*Campylobacter*	1.11 (1.06)	1.54 (0.98)	0.190
*Granulicatella*	0.03 (0.04)	0.14 (0.18)	0.008
*Cloacibacterium*	0.01 (0.03)	0.05 (0.09)	0.038
*Lautropia*	1.69 (2.88)	1.15 (1.24)	0.416
*Abiotrophia defective*	0.26 (0.40)	0.05 (0.10)	0.067
*Eikenella corrodens*	0.19 (0.41)	0.25 (0.35)	0.619
*Shaalia odontolytica*	0.46 (0.54)	0.78 (0.83)	0.172
*Leptotricha*	1.96 (3.59)	1.13 (1.77)	0.338
*Stomatobaculum longum*	0.77 (0.91)	0.94 (1.20)	0.635
*Granucicatella elegans*	0.11 (0.29)	0.09 (0.27)	0.818
*Tannerella*	0.11 (0.17)	0.42 (0.66)	0.034
*Pasteurellaceae*	0.48 (0.58)	0.28 (0.55)	0.301
*Bifidobacteriaceae*	0.11 (0.17)	0.15 (0.20)	0.539
*Atopobium*	0.08 (0.16)	0.08 (0.23)	0.899
*Oribacterium*	0.11 (0.32)	0.04 (0.12)	0.350
*Enterobacteriaceae*	1.93 (2.37)	1.79 (1.78)	0.835
*Parascardovia denticolens*	0.02 (0.08)	0.05 (0.13)	0.462
*Butyrivibrio*	0.03 (0.08)	0.01 (0.03)	0.383
*Eikenella*	0.25 (0.38)	0.45 (0.87)	0.340
*Cardiobacterium hominis*	0.09 (0.30)	0.03 (0.04)	0.422
*Bergeyella cardium*	0.43 (0.81)	0.04 (0.09)	0.075
*Catonella*	0.09 (0.12)	0.21 (0.48)	0.328
*Vallitalae okinawensis*	0.02 (0.04)	0.01 (0.03)	0.599
*Olsenella*	0.13 (0.40)	0.18 (0.50)	0.751
*Mogibacterium*	0.06 (0.12)	0.03 (0.05)	0.384
*Eubacterium*	0.23 (0.43)	0.18 (0.53)	0.793
*Sulfurihydrogenibium*	0.18 (0.52)	0.03 (0.05)	0.253
*Finegoldia*	0.06 (0.09)	0.01 (0.03)	0.030

**Table 13:** Risk of radiotherapy complications in patient groups with different levels of *Porphyromonas* and *Fusobacterium* bacteria in the microbiome, significance test results, odds ratio values, and their 95% confidence intervals.

Genus	RIOM—Radiation-Induced Oral Mucositis		*p*-Value	OR (95% CI)
	Yes	No		
*Porphyromonas* ≥ 2.9%	16 (100.0%)	1 (4.2%)	<0.001	1209 (15.8–92801)
*Porphyromonas* < 2.9%	0 (0.0%)	23 (95.8%)		1.00 (ref.)
*Fusobacterium* ≥ 3.2%	16 (100.0%)	5 (20.8%)	<0.001	238 (4.09–13903)
*Fusobacterium* < 3.2%	0 (0.0%)	19 (79.2%)		1.00 (ref.)

**Table 14:** Descriptive statistics of the percentage of identified bacteria and fungi in the study and control groups and results of significance tests (Welch’s *t*-test).

Culture Result—Phylum (%)	RIOM—Radiation-Induced Oral Mucositis		*p*-Value
	Yes N = 16	No N = 24	
*Bacteroidetes*	27.84 (6.70)	28.29 (5.93)	0.825
*Bacteroidota*	8.38 (4.05)	3.94 (2.41)	0.001
*Fusobacteriota*	7.69 (4.41)	3.00 (2.75)	0.001
*Proteobacteria*	5.77 (2.84)	7.34 (3.22)	0.121
*Firmicutes*	18.62 (4.66)	22.53 (5.56)	0.026
*Baccillota*	3.31 (3.41)	2.33 (1.90)	0.578
*Actinomycetota*	3.81 (2.83)	5.36 (2.08)	0.054
*Pseudomonadota*	2.86 (3.41)	2.33 (1.90)	0.578
*Actinobacteria*	14.70 (4.87)	15.82 (5.72)	0.525
*Spirochaetes*	0.04 (0.09)	0.08 (0.17)	0.317
*Candidatus*	0.12 (0.14)	0.13 (0.16)	0.901

**Table 15:** General characteristics of patients in groups with different survival rate and results of significance and independence tests.

Variable	Survival Rate (Months)				*p*-Value
	12–24 N = 6	24–36 N = 8	36–48 N = 2	>48 N = 24	
Gender:					0.672
Male. *n* (%)	4 (66.7)	7 (87.5)	1 (50.0)	17 (70.8)	
Female. *n* (%)	2 (33.3)	1 (12.5)	1 (12.5)	7 (29.2)	
Age (years):					0.658
*M* (*SD*)	65.3 (6.2)	65.4 (6.6)	68.0 (29.7)	62.1 (8.3)	
Education:					0.190
Primary	1 (16.7)	0 (0.0)	0 (0.0)	0 (0.0)	
Secondary	2 (33.3)	6 (75.0)	1 (50.0)	9 (37.5)	
Incomplete higher	2 (33.3)	2 (25.0)	1 (50.0)	6 (25.0)	
Higher	1 (16.7)	0 (0.0)	0 (0.0)	9 (37.5)	
Place of residence:					0.286
Village	2 (33.3)	2 (25.0)	0 (0.0)	4 (16.7)	
Town up to 20,000	1 (16.7)	3 (37.5)	0 (0.0)	3 (12.5)	
21,000–50,000 inhabitants	0 (0.0)	0 (0.0)	0 (0.0)	8 (33.3)	
Over 50,000	3 (50.0)	3 (37.5)	2 (100.0)	9 (37.5)	
Economic zone/urban area:					0.846
Yes	5 (83.3)	6 (75.0)	2 (100.0)	18 (75.0)	
No	1 (16.7)	2 (25.0)	0 (0.0)	6 (25.0)	
Marital status:					0.542
Single	1 (16.7)	1 (12.5)	0 (0.0)	0 (0.0)	
In a relationship	4 (66.6)	6 (75.0)	2 (100.0)	18 (75.0)	
With family support	1 (16.7)	1 (12.5)	0 (0.0)	6 (25.0)	
BMI (kg/m^2^):					0.423
Mean (SD)	24.7 (2.8)	23.6 (1.2)	22.5 (0.0)	24.1 (1.7)	

**Table 16:** General characteristics of patients in groups with different survival rate and results of significance and independence tests.

Variable	Survival Rate (Months)				*p*-Value
	12–24 N = 6	24–36 N = 8	36–48 N = 2	>48 N = 24	
Hemoglobin (g/dL)	13.3 [12.4; 14.8]	13.5 [12.5; 14.1]	11.9 [11.5; 12.3]	13.3 [11.5; 14.1]	0.721
Leukocytes (×10^3^/mm)	7.6 [5.2; 8.4]	8.1 [6.7; 9.2]	9.0 [8.7; 9.4]	7.5 [7.0; 8.6]	0.595
Total protein (×10^3^/μL)	6.8 [6.4; 7.1]	6.4 [6.3; 6.8]	6.9 [6.9; 7.1]	7.0 [6.7; 7.2]	0.023
CRP (mg/L)	6.5 [5.0; 8.7]	6.3 [5.6; 7.2]	5.5 [4.1; 7.0]	4.1 [3.3; 4.9]	0.027
ECOG scale (score):					0.861
Asymptomatic	5 (83.3)	6 (75.0)	2 (100.0)	20 (83.3)	
Completely ambulatory	1 (16.7)	2 (25.0)	0 (0.0)	4 (16.7)	
Chronic diseases (yes)	3 (50.0)	4 (50.0)	1 (50.0)	12 (50.0)	1.000
Arterial hypertension, cardiovascular diseases	3 (50.0)	4 (50.0)	1 (50.0)	12 (50.0)	1.000
Diabetes	1 (16.7)	0 (0.0)	0 (0.0)	0 (0.0)	0.121
Lower respiratory tract diseases	1 (16.7)	4 (50.0)	0 (0.0)	2 (8.3)	0.053
Smoking	5 (83.3)	5 (62.5)	1 (50.0)	12 (50.0)	0.509
Drinking alcohol regularly	0 (0.0)	1 (12.5)	0 (0.0)	4 (16.7)	0.677
Dental condition:					0.027
1—Normal	2 (33.3)	0 (0.0)	0 (0.0)	13 (54.2)	
2—Cavities, caries, periodontal disease	4 (66.7)	7 (87.5)	2 (100.0)	6 (25.0)	
3—Edentulism	0 (0.0)	1 (12.5)	0 (0.0)	5 (20.8)	
VAS (score)					0.073
0	4 (66.7)	8 (100.0)	2 (100.0)	23 (95.8)	
1	2 (33.3)	0 (0.0)	0 (0.0)	1 (4.2)	
Positive culture result	4 (66.7)	6 (75.0)	2 (100.0)	2 (8.3)	<0.001
HPV+/p16+:					0.350
CUP	0 (0.0)	1 (12.5)	1 (50.0)	6 (25.0)	
Non-viral related CUP	6 (100.0)	7 (87.5)	1 (50.0)	18 (75.0)	
Mucitis in course of radiotherapy	5 (83.3)	8 (100.0)	2 (100.0)	1 (4.2)	<0.001

**Table 17:** Basic statistics (Me and IQR) of the proportions of isolated bacteria in groups of patients with different survival rates and the results of the analysis of variance.

Culture Result—Genus (%)	Survival Rate (Months)				ANOVA *p*-Value
	12–24	24–36	36–48	48+	
*Streptococcus Oralis*	15.3 (3.4)	17.3 (5.9)	12.6 (14.7)	21.5 (4.0)	0.012
*Prevotella*	27.2 (16.3)	28.7 (9.2)	27.5 (8.7)	29.4 (8.3)	0.956
*Rothia micilaginosa*	2.5 (3.6)	1.3 (3.6)	1.8 (0.2)	4.1 (3.0)	0.108
*Aggregatibacter*	1.2 (0.5)	2.2 (1.9)	0.5 (0.4)	2.1 (1.7)	0.341
*Gemella*	0.5 (0.9)	0.7 (1.9)	0.6 (0.2)	1.5 (1.7)	0.300
*Porphyromonas*	3.3 (2.0)	4.7 (4.1)	7.2 (2.1)	0.9 (1.2)	<0.001
*Fusobacterium*	3.9 (2.9)	6.0 (4.0)	5.2 (4.0)	1.4 (2.7)	<0.001
*Corynebacterium matruch.*	1.0 (1.2)	1.3 (1.9)	0.9 (1.7)	1.5 (1.9)	0.531
*Neiseria*	2.4 (4.4)	2.8 (1.5)	2.6 (3.8)	2.8 (2.0)	0.950
*Kingella*	0.8 (1.0)	0.1 (1.2)	0.6 (1.2)	0.9 (1.1)	0.495
*Lacto bacillales*	0.3 (0.4)	0.2 (0.4)	0.9 (1.2)	0.1 (0.2)	0.139
*Actinobacteria*	7.1 (2.2)	6.0 (6.1)	8.1 (3.3)	7.9 (5.7)	0.896
*Actinomyces*	9.4 (6.8)	5.5 (3.0)	3.0 (5.9)	4.2 (4.5)	0.059
*Haemophilus*	3.1 (2.8)	1.6 (2.0)	2.6 (2.7)	3.1 (1.7)	0.071
*Capnocytophaga gr/gi.*	2.8 (2.5)	0.8 (3.1)	1.4 (1.8)	2.0 (2.2)	0.259
*Treponema*	0.0 (0.0)	0.0 (0.1)	0.0 (0.0)	0.0 (0.1)	0.732
*Candidadus Sacharimonas*	0.2 (0.2)	0.1 (0.3)	0.0 (0.0)	0.1 (0.2)	0.468
*Clostridiales*	0.5 (0.7)	0.6 (0.6)	0.1 (0.0)	0.6 (1.2)	0.439
*Veilonella*	0.0 (0.0)	0.0 (0.7)	0.3 (0.5)	0.3 (1.2)	0.368
*Campylobacter*	1.4 (1.6)	0.6 (2.5)	1.1 (1.8)	1.5 (1.4)	0.692
*Granulicatella*	0.0 (0.1)	0.0 (0.0)	0.0 (0.0)	0.1 (0.2)	0.047
*Cloacibacterium*	0.0 (0.0)	0.0 (0.0)	0.0 (0.0)	0.0 (0.1)	0.226
*Lautropia*	1.2 (0.4)	0.6 (2.0)	6.0 (12.0)	0.8 (2.0)	0.879
*Abiotrophia defective*	0.1 (0.1)	0.0 (0.5)	0.3 (0.6)	0.0 (0.1)	0.741
*Eikenella corrodens*	0.4 (0.9)	0.0 (0.2)	0.0 (0.0)	0.1 (0.3)	0.289
*Shaalia odontolytica*	0.4 (0.7)	0.2 (0.6)	0.4 (0.8)	0.5 (1.1)	0.755
*Leptotricha*	0.8 (2.6)	0.2 (3.3)	0.7 (0.1)	0.7 (1.3)	0.977
*Stomatobaculum longum*	0.8 (1.7)	0.2 (0.8)	0.6 (1.2)	0.2 (1.5)	0.355
*Granucicatella elegans*	0.0 (0.0)	0.0 (0.0)	0.9 (0.1)	0.0 (0.0)	0.010
*Tannerella*	0.1 (0.2)	0.0 (0.4)	0.0 (0.0)	0.2 (0.5)	0.225
*Pasteurellaceae*	0.4 (0.6)	0.3 (0.6)	1.0 (1.6)	0.1 (0.2)	0.297
*Bifidobacteriaceae*	0.1 (0.4)	0.1 (0.1)	0.0 (0.0)	0.1 (0.2)	0.406
*Atopobium*	0.0 (0.0)	0.0 (0.2)	0.1 (0.2)	0.0 (0.1)	0.671
*Oribacterium*	0.0 (0.1)	0.0 (0.0)	0.7 (1.3)	0.0 (0.0)	0.450
*Enterobacteriaceae*	1.4 (2.2)	2.9 (2.6)	0.0 (0.0)	1.2 (2.3)	0.123
*Parascardovia denticolens*	0.0 (0.0)	0.0 (0.0)	0.0 (0.0)	0.0 (0.0)	0.695
*Butyrivibrio*	0.0 (0.0)	0.0 (0.1)	0.0 (0.0)	0.0 (0.0)	0.737
*Eikenella*	0.3 (1.1)	0.1 (0.4)	0.0 (0.0)	0.0 (0.2)	0.168
*Cardiobacterium hominis*	0.0 (0.0)	0.0 (0.1)	0.0 (0.0)	0.0 (0.1)	0.474
*Bergeyella cardium*	0.1 (0.3)	0.0 (0.3)	1.1 (2.2)	0.0 (0.0)	0.096
*Catonella*	0.1 (0.1)	0.1 (0.2)	0.0 (0.0)	0.0 (0.2)	0.303

**Table 18:** Descriptive statistics of the percentage of identified bacteria and fungi in the study and control groups and results of significance tests (Kruskal–Wallis sum rank test).

Culture Result—Phylum (%)	Survival Rate (Months)				Kruskal–Wallis Test *p*-Value
	12–24 N = 6	24–36 N = 8	36–48 N = 2	48+ N = 24	
*Bacteroidetes*	27.2 [16.3]	28.7 [9.2]	27.5 [8.7]	29.4 [8.3]	0.956
*Bacteroidota*	8.3 [6.7]	6.4 [4.3]	9.9 [3.0]	3.6 [2.1]	<0.001
*Fusobacteriota*	5.7 [2.3]	8.3 [4.2]	6.1 [3.7]	2.9 [3.8]	0.002
*Proteobacteria*	7.2 [5.6]	4.9 [5.8]	4.5 [2.5]	6.7 [3.4]	0.343
*Firmicutes*	16.8 [2.7]	19.0 [4.9]	14.6 [13.8]	23.4 [4.5]	0.006
*Baccillota*	3.1 [2.0]	2.3 [2.1]	7.0 [1.4]	3.2 [2.5]	0.115
*Actinomycetota*	3.4 [4.1]	2.2 [3.7]	3.5 [0.0]	5.5 [2.6]	0.076
*Pseudomonadota*	2.2 [0.8]	2.3 [1.9]	7.6 [14.8]	2.3 [1.8]	0.992
*Actinobacteria*	17.5 [4.2]	12.7 [4.4]	11.9 [0.9]	14.7 [4.3]	0.123
*Spirochaetes*	0.0 [0.0]	0.0 [0.1]	0.0 [0.0]	0.0 [0.1]	0.732
*Candidatus*	0.2 [0.2]	0.1 [0.3]	0.0 [0.0]	0.1 [0.2]	0.468

Me [IQR], IQR = Q3–Q1.

## Data Availability

The raw data supporting the conclusions of this article will be made available by the authors on request.
